# 3D printed electrodes for efficient membrane capacitive deionization†

**DOI:** 10.1039/c9na00507b

**Published:** 2019-10-08

**Authors:** Sareh Vafakhah, Glenn Joey Sim, Mohsen Saeedikhani, Xiaoxia Li, Pablo Valdivia y Alvarado, Hui Ying Yang

**Affiliations:** Pillar of Engineering Product Development, Singapore University of Technology and Design Singapore 487372 yanghuiying@sutd.edu.sg; Department of Materials Science and Engineering, National University of Singapore 9 Engineering Drive 1 Singapore 117576; Beijing Advanced Innovation Centre for Biomedical Engineering, School of Chemistry, Beihang University Beijing 100191 P. R. China

## Abstract

There is increasing interests in cost-effective and energy-efficient technologies for the desalination of salt water. However, the challenge in the scalability of the suitable compositions of electrodes has significantly hindered the development of capacitive deionization (CDI) as a promising technology for the desalination of brackish water. Herein, we introduced a 3D printing technology as a new route to fabricate electrodes with adjustable composition, which exhibited large-scale applications as free-standing, binder-free, and robust electrodes. The 3D printed electrodes were designed with ordered macro-channels that facilitated effective ion diffusion. The high salt removal capacity of 75 mg g^−1^ was achieved for membrane capacitive deionization (MCDI) using 3D printed nitrogen-doped graphene oxide/carbon nanotube electrodes with the total electrode mass of 20 mg. The improved mechanical stability and strong bonding of the chemical components in the electrodes allowed a long cycle lifetime for the MCDI devices. The adjusted operational mode (current density) enabled a low energy consumption of 0.331 W h g^−1^ and high energy recovery of ∼27%. Furthermore, the results obtained from the finite element simulations of the ion diffusion behavior quantified the structure–function relationships of the MCDI electrodes.

## Abstract 2

3D printed electrodes for cost-effective and energy-efficient desalination technologies.

## Graphical abstract

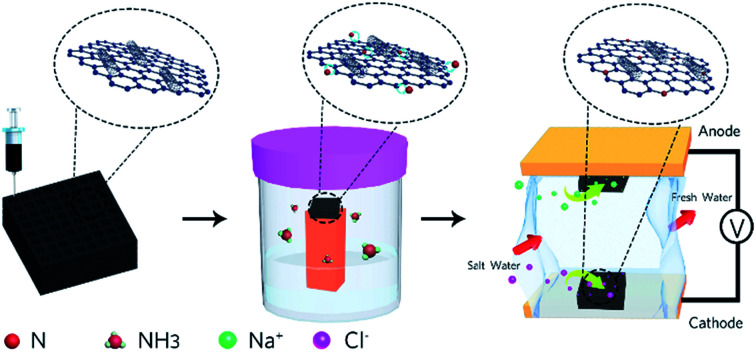

## Introduction

Capacitive deionization (CDI), a promising electrochemical technology, has attracted significant attention for the desalination of brackish water. In addition, more recently, alternative concepts such as membrane capacitive deionization (MCDI),^1–3^ flow electrode CDI (FCDI),^4,5^ flow-through electrode CDI (FtCDI),^6,7^ and hybrid CDI (HCDI)^8,9^ have been explored to improve the production of water and salt removal capacities. Generally, their fundamental working mechanism is based on driving sodium and chloride ions to the interior of oppositely charged electrodes during the reversible process. Among the different CDI-based technologies, MCDI is able to achieve higher desalination efficiency by eliminating the co-ion repulsion effects.^2^ The final performance and the related mechanisms of ion storage are highly dependent on the electrode materials, which can be grouped as carbon-based (including activated carbon (AC),^10^ carbon nanotubes (CNTs),^6,11^ carbon-coated particles, and graphene^12^) and redox-active materials (such as Prussian blue analogues,^8,9^ Na_3_V_2_(PO_4_)_3_,^13^ NaTi_2_(PO_4_)_3_,^14^ Na_0.44_MnO_2_,^15^ and Na_1.1_V_3_O_7.9_ (ref. 16)). Typically, the MCDI electrodes are prepared by coating the current collectors with the slurry of a mixture of active materials, conductive additives, and polymer binders through different coating technologies. To maximize the efficiency of MCDI, the as-prepared electrodes should have the required functionality without any insulating components; unfortunately, the application of most of the preparation methods for the current electrodes is hindered by the major drawbacks such as the use of polymer binders, which may block the pathway for ions and increase the internal resistance of the electrodes, severely limiting the salt removal capacity.^17–19^ However, a radical improvement in electrode performance might be achieved if various free-standing three-dimensional (3D) structures could be developed *via* alternative ways. To date, several routes have been proposed to prepare 3D structures such as carbon aerogels,^20^ graphene aerogels,^12,21^ carbon nanotube/graphene hybrid sponges,^11^ activated carbon nanofibers,^10,22,23^ and redox-active nanoparticles/graphene aerogels.^8,24^ In this regard, the most common chemical and physical strategies include direct drying, self-assembly of gel-based materials *via* hydrothermal reduction, and template-guided methods such as coating metallic foam using chemical vapor deposition (CVD).^25,26^ Although these 3D structures possess relatively better performance due to the interconnected pores, which improve conductivity and electrolyte ion diffusion, they still do not meet the requirements for practical application. Usually, these electrodes are not able to achieve optimum mechanical stability and cost-effective large-scale deployment.^25^ In addition, their network architecture is very random and incidental, which may limit their further applications.^25,27,28^ In this regard, the key point is to achieve a controllable and scalable method to assemble the desired structure while maintaining the characteristic properties of materials. Therefore, it is indeed of utmost importance to develop a systematic strategy to combine the active materials using a controllable electrode production method.

Herein, we demonstrated an experimental process for the construction of a 3D free-standing electrode by the 3D printing technology. Among carbon materials, graphene oxide (GO) was chosen as a desirable electrode precursor due to its outstanding properties such as large surface area with a broad range of pore morphologies, high conductivity, good mechanical stability, simple synthesis procedure, and ability to self-stand.^12,29,30^ As an electrode production method, the 3D printing technology was proposed as a feasible strategy to improve the tunable fabrication of graphene macrostructures for MCDI (and its analogues) electrodes. The main challenge in the fabrication of 3D printable electrodes for MCDI is how to prepare an ink with suitable viscosity that shows shear-thinning behavior while retaining the necessary properties such as good electrochemical behavior and electrical conductivity.^25,31^ Graphene sheets are known to be very likely to restack together due to their strong π–π interactions, which make it difficult for the ions to penetrate the inner layer of graphene.^32^ As a result, functional additives are necessary to improve the final electrochemical performance as they enhance the electrical conductivity and the number of accessible pores along with optimizing the ink viscosity.^27,29,33,34^ Among the functional additives, one-dimensional carbon nanotubes (CNTs) are an ideal choice for this goal.

**Fig. 1:**
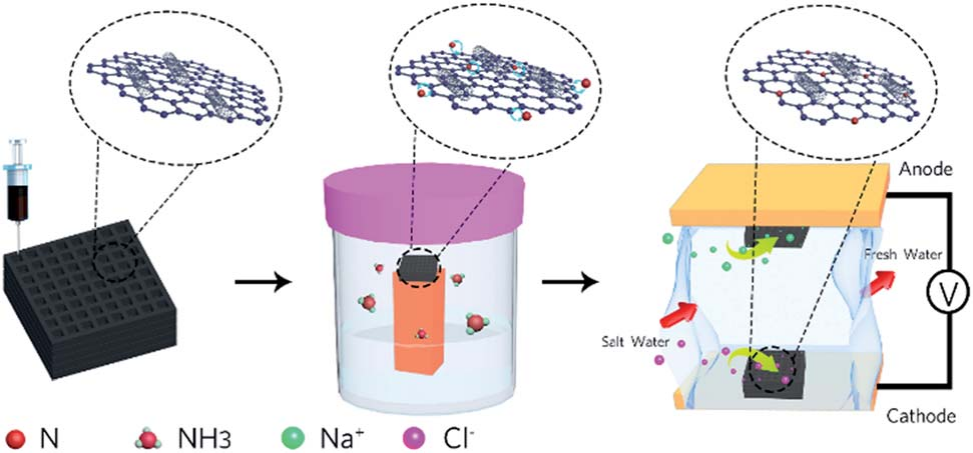
Schematic of the preparation process for the electrodes and the MCDI device.

Previously, a few studies have been reported on the fabrication of graphene macro-architectures using the 3D printing technology; however, there is still no systematic study that evaluates the application of 3D printed electrodes in MCDI. Herein, the application of a nitrogen-doped GO/CNT macrostructure as a symmetric electrode in MCDI was successfully explored. The schematic of the preparation of the electrodes is shown in Fig. 1, and the images of the 3D printed GO/CNT with different numbers of layers are shown in Fig. S1 (ESI†). Moreover, an optimum precursor was selected by adjusting the GO to CNT (as conductive and viscosity agents) ratio, respectively. The final free-standing electrodes illustrated the successful removal of salt ions within the MCDI device assemblies.

## Results and discussion

### Characterization

**Fig. 2:**
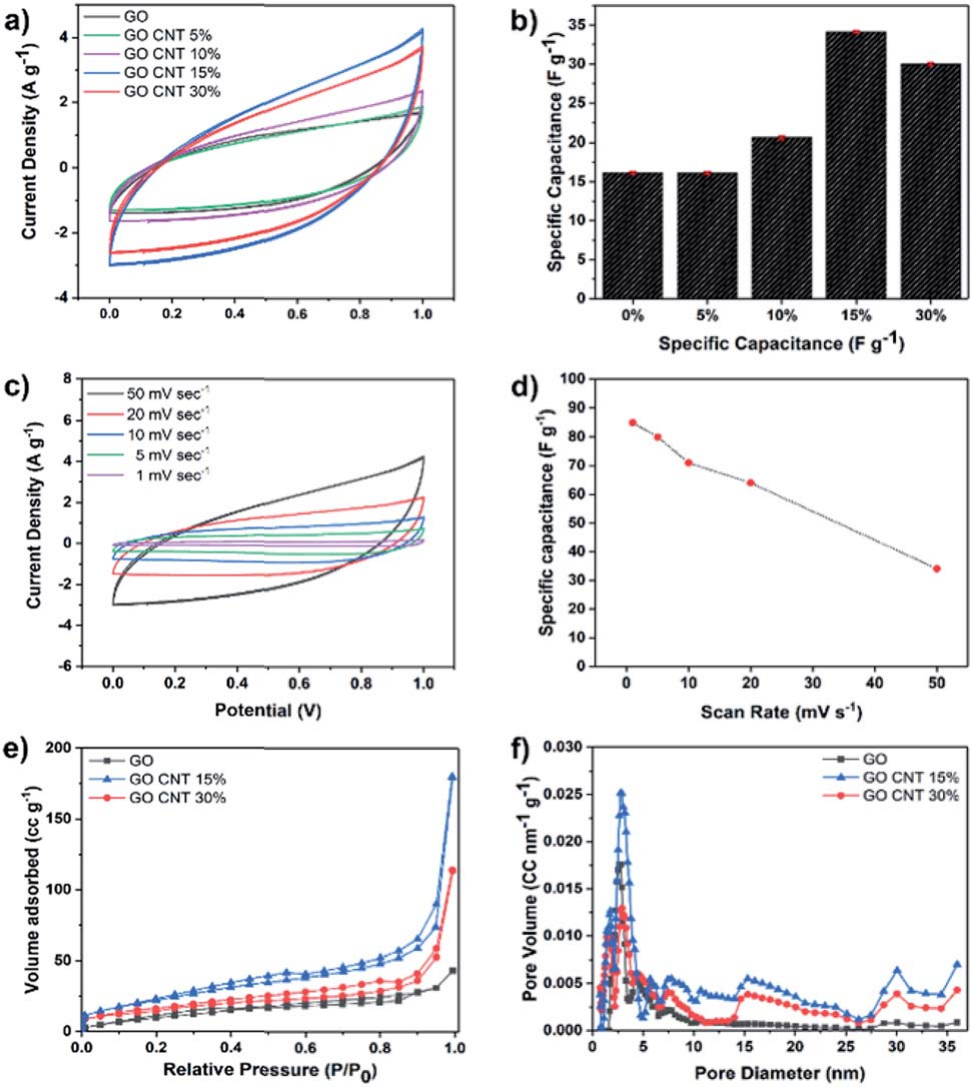
(a) CV comparison and (b) the corresponding specific capacitance of GO and GO/CNT ink with different ratios of 95 : 5, 90 : 10, 85 : 15, and 70 : 30. (c) CV at different scan rates for GO/CNT 15% ink. (d) Specific capacitances at the corresponding scan rates. (e) The BET analysis and (f) the porosity distribution based on the DFT method for GO and GO/CNT (15% and 30%).

Cyclic voltammetry (CV) measurements were carried out in the potential sweep range from 0 to 1 V (*vs.* Ag/AgCl) at the scan rate of 50 mV s^−1^ to compare the total electrosorption capacity of the inks with different compositions. Fig. 2(a) illustrates the results of the CV measurements for GO and GO/CNT inks with different ratios of the components, and the influence of the CNT was fully investigated. It is clearly shown that they all possess the typical capacitor-like behaviour with no oxidation/reduction reactions; this suggests electrical double-layer behaviour as the main mechanism of ion adsorption on the surface of the electrodes.^19,35^ Then, the specific capacitance of all electrodes was calculated, as shown in Fig. 2(b). Note that the relatively low specific capacitances of the electrodes can be attributed to the high scan rate, and more importantly, the ink precursors have not been reduced (GO/CNT without any conductive additives). Moreover, the goal of the experiments in this step was to figure out the best ratio of GO to CNT for further experiments. As can be observed in Fig. 2(b), all the ink precursors with CNTs show higher specific capacitance than pure GO. Moreover, the specific capacitance was initially improved by increasing the amount of CNT in the slurry composite (34.1 F g^−1^ for the GO CNT 15%); however, the value decreased with a further increase in the CNT content from 15% to 30%, as obviously seen in Fig. 2(b). The improvement in the capacitance is ascribed to the formation of a conductive network between GO and CNT, which efficiently generates conductive channels in the electrodes.^36,37^ In addition, CV experiments were conducted for the GO CNT 15% at the different scan rates of 1, 5, 10, 20, and 50 mV s^−1^, as presented in Fig. 2(c). At higher scan rates, a leaf-like shape of the CV curves is more obvious; however, the distortion gradually becomes weaker with a decrease in the scan rate from 50 mV s^−1^ to 1 mV s^−1^ due to an increase in the ion transportation rate. Moreover, as shown in Fig. 2(d), the specific capacitance of the ink with the composition GO CNT 15% could be increased up to around 90 F g^−1^ for the lower scan rate of 1 mV s^−1^. To further explain the decrease in capacitance with an increase in the CNT contents from 15% to 30%, the surface areas and the pore distributions of the electrodes (GO and GO : CNT with the ratios of 85 : 15 and 70 : 30) were studied. The results are shown in Fig. 2(e) and (f).

As illustrated in Fig. 2(e), all the isotherms exhibit a typical type IV hysteresis loop according to the AUPAC classification, implying the presence of a mesoporous structure; as shown in Fig. 2(e), largest nitrogen uptake has been obtained for GO/CNT 15% (surface area of 83.6 m^2^ g^−1^*vs.* 55.16 m^2^ g^−1^ for GO/CNT 30%), which is also significantly larger than that of pure GO with the surface area of 40 m^2^ g^−1^. These results illustrate that CNTs in an optimized amount effectively prevent the aggregation of GO sheets and improve the surface area and the corresponding specific capacitance; however, CNTs at higher percentages aggregate and cover the GO sheets and lead to a decline in the electrosorption performance. As a result, the ink slurry with the composition GO/CNT 15% was chosen for further experiments. As depicted in Fig. 2(f), the density functional theory (DFT) pore size distribution indicates the presence of mesopores with the average pore diameters of 2.7 nm and 7.9 nm for GO, GO/CNT 15% and 30%. Moreover, the introduction of CNTs increases the percentage of mesopores with the sizes 15, 30 and 36 nm, which will be favorable for the capacitive performance.^11^ In brief, the introduction of an efficient combination of micropores, mesopores and macropores (*via* 3D printing) is the main key to further enhance the salt removal capacity of the electrodes.^38^

**Fig. 3:**
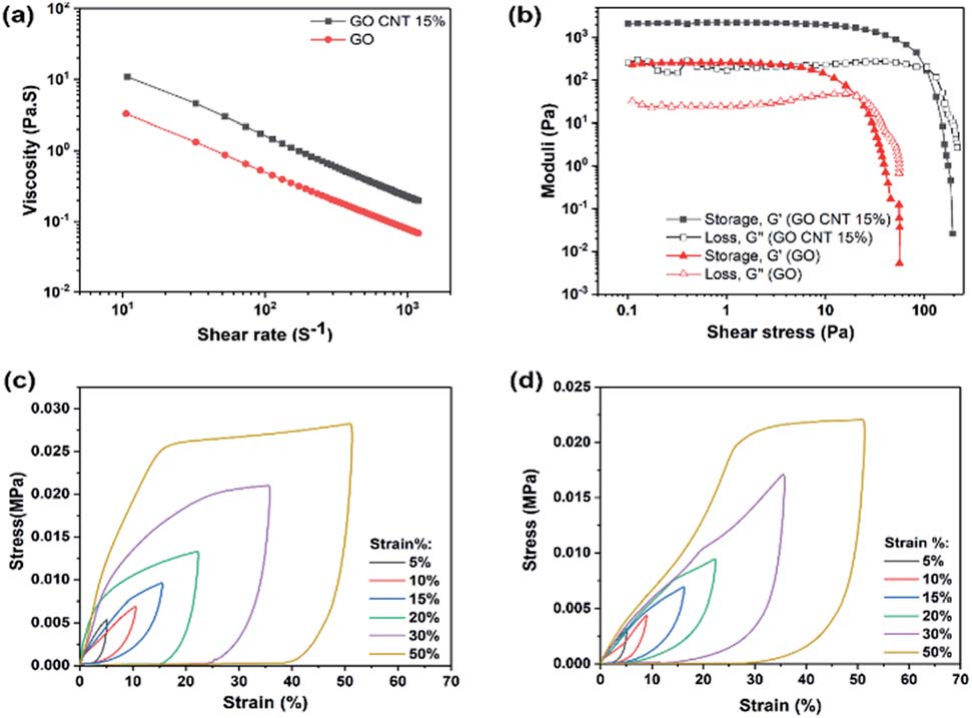
Mechanical properties of GO and GO/CNT inks: (a) log–log curve for the viscosity as a function of shear rate and (b) storage and loss modulus *vs.* shear stress. Stress–strain curves of (c) 3D printed GO and (d) GO/CNT 15%.

To achieve an ideal 3D printed structure that can easily flow through the fine nozzle and retain its geometry after deposition, the rheological behavior of the prepared ink is of high priority. Fig. 3(a) illustrates the apparent viscosity of the concentrated inks as a function of shear rate, indicating higher viscosity of GO/CNT as compared to that of GO.

In addition, note that both inks demonstrate shear-thinning non-Newtonian behavior, which is necessary for their extrusion during the printing process. However, the GO/CNT ink with higher viscosity, originated from the optimum amount of CNT, is more favorable for bearing the top layer weight and retaining the overall geometry;^39^ for comparing the viscoelastic behaviors of the inks, the storage and loss modulus (*G*′ and *G*′′) were investigated as the shear stress variables, and the results are displayed in Fig. 3(b). The viscoelastic plateau value increased from ∼200 to 2000 Pa upon the introduction of the CNTs into the ink composition, and the shear yield stress (*G*′ = *G*′′) was enhanced from *τ y* = 20 Pa to 100 Pa (indicating a stiffer nature of the GO/CNT as compared to that of GO). For both inks, initially, the storage modulus was higher than the loss modulus (*G*′ > *G*′′); this indicates the inherent solid behavior, and it represents the linear viscoelastic region.^31,39^ Upon increasing the shear stress, the inherent liquid behavior becomes dominant until the liquid starts flowing at the yield point, which eases the flow through the nozzles. It can be concluded that both inks are suitable for the bottom-up layer-by-layer printing. However, well-dispersed CNTs act as conductivity agents as well as improve the printability.^28,40^ Furthermore, as illustrated in Fig. 3(c) and (d), the mechanical robustness of the 3D printed structures was quantified by an in-plane compression test using a dynamic mechanical analyzer (DMA). Both figure (c) and (d) display the compression behavior of the 3D printed macrostructures as a function of the predefined strain percentage for GO and GO/CNT, respectively. As observed in the figures, both GO and GO/CNT show reversible non-linear super-elastic compressibility up to strains as high as 50%, indicating good deformation memory effects. Note that the maximum stress for all the strain percentages is slightly lower in GO/CNT as compared to that in the GO macrostructure. This may be ascribed to the elimination of some π–π bonds from the GO sheets as a result of the insertion of CNTs into the system; these π–π bonds between the sheets are highly effective for distributing the load across the entire macrostructure, which are partially substituted by van der Waals interactions between GO and CNTs in the GO/CNT macrostructures.^41,42^

**Fig. 4:**
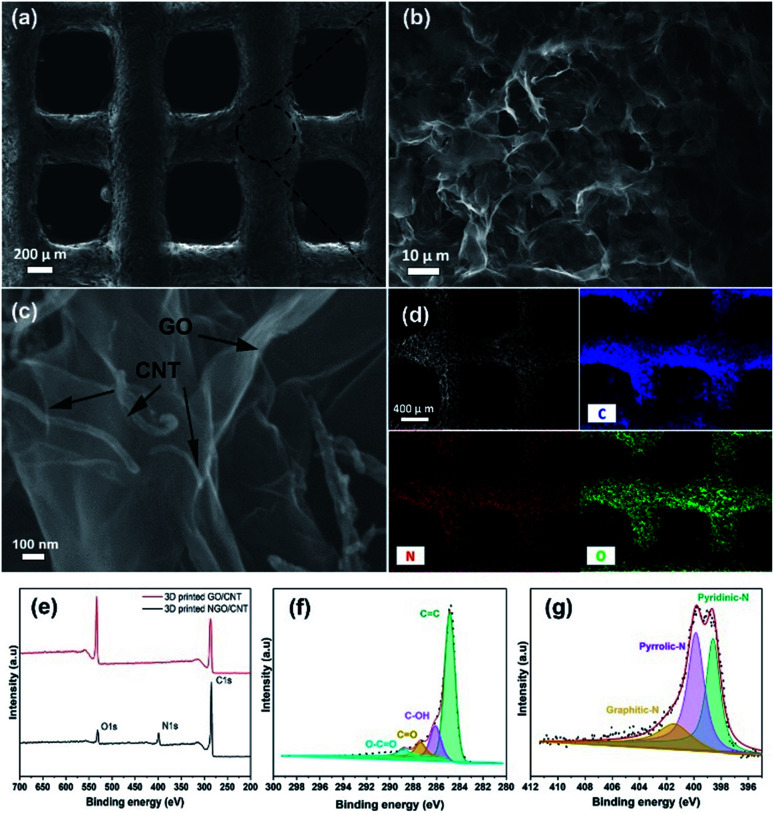
(a)–(c) SEM images of 3D printed GO/CNTs at different resolutions; (d) EDS mapping obtained after nitrogen doping. (e) The XPS full spectrum of 3D printed GO/CNTs before and after the nitrogen doping process. (f) The carbon survey and (g) the nitrogen survey spectra for the 3D printed NGO/CNTs.

The investigation of the morphology of the 3D printed GO/CNTs was conducted using scanning electron microscopy (SEM), as shown in Fig. 4(a)–(c); in Fig. 4(a), the excellent accuracy and integrity of the cube-like structure is noticeable, which is a result of good ink preparation and proper adjustment of the 3D printing process. Moreover, the higher-magnification SEM image of the porous interconnected structure is shown in Fig. 4(b) and (c) shows the contents of the CNTs distributed along with GO sheets that improve the conductivity and increase the available pore sites; furthermore, the presence of carbon and oxygen and the successful doping of nitrogen into the structure have been confirmed by energy dispersive X-ray (EDX) mapping of the electrode (Fig. 4(d)). To further study the chemical states and the atomic ratios of the 3D printed GO/CNT samples before and after the solid/gas interface hydrothermal reaction, X-ray photoelectron spectroscopy (XPS) was carried out, and the results are presented in Fig. 4(e)–(g). For both samples, there are two peaks attributed to C 1s and O 1s (as shown in Fig. 4(e)). In addition, after nitrogen doping, a new peak appears, which is assigned to N 1s, and the existing O 1s peak weakens accordingly. The oxygen content decreased from around 26 to 7 at%, indicating successful reduction. Moreover, the high-resolution spectrum of C 1s (as shown in Fig. 4(f)) for the N-doped GO/CNT reveals four peaks located at 284.8 eV, 286.1 eV, 287.3 eV, and 288.8 eV, which correspond to C=C, C–OH, C=O, and O–C=O configurations, respectively. In addition, the N 1s spectrum was investigated to study the content and configuration of doped nitrogen. As shown in Fig. 4(g), the N 1s peak is deconvoluted into three main peaks: graphitic N (400.7 eV), pyrrolic N (399.8 eV), and pyridinic N (398.62 eV). Pyridinic-N indicates the doping amount of nitrogen at the edge of the GO sheets or at the edge of the defects, whereas pyrrolic N shows the presence of amides (C(O)–NH_2_) or amines (–NH_2_) in the GO sheets. Moreover, finally, graphitic N is ascribed to the nitrogen atoms that substitute one carbon atom and bind to other three carbon atoms, which later contribute to the enhancement in conductivity.^43–46^ The C 1s spectrum obtained before the nitrogen doping process is presented in Fig. S2.†

The results obtained from XPS are in agreement with the Raman spectra shown in Fig. S3.† The Raman spectra obtained for the 3D printed GO/CNT and N-doped GO/CNT illustrate typical peaks (D band and G band) located at around 1370 cm^−1^ and 1620 cm^−1^.^29,47^ The intensity ratio of the D band to the G band (*I*_D_/*I*_G_) is generally used to evaluate the impact of the reduction process. As shown in Fig. S3,† the average ratio increased from 0.96 for the pristine GO/CNT to 1.04 for the N-doped GO/CNT. The result demonstrates an increase in the sp^2^ domain, which ensures the efficient reduction of GO/CNTs.^43,44^ In addition, the individual conductivities of the electrodes were measured using the four-point probe station at room temperature, and the conductivities of 34 S m^−1^, 50 S m^−1^, and 150 S m^−1^ were obtained for GO, GO/CNT and N-doped GO/CNT, respectively.

### Desalination properties of 3D printed N-doped GO/CNTs

**Fig. 5:**
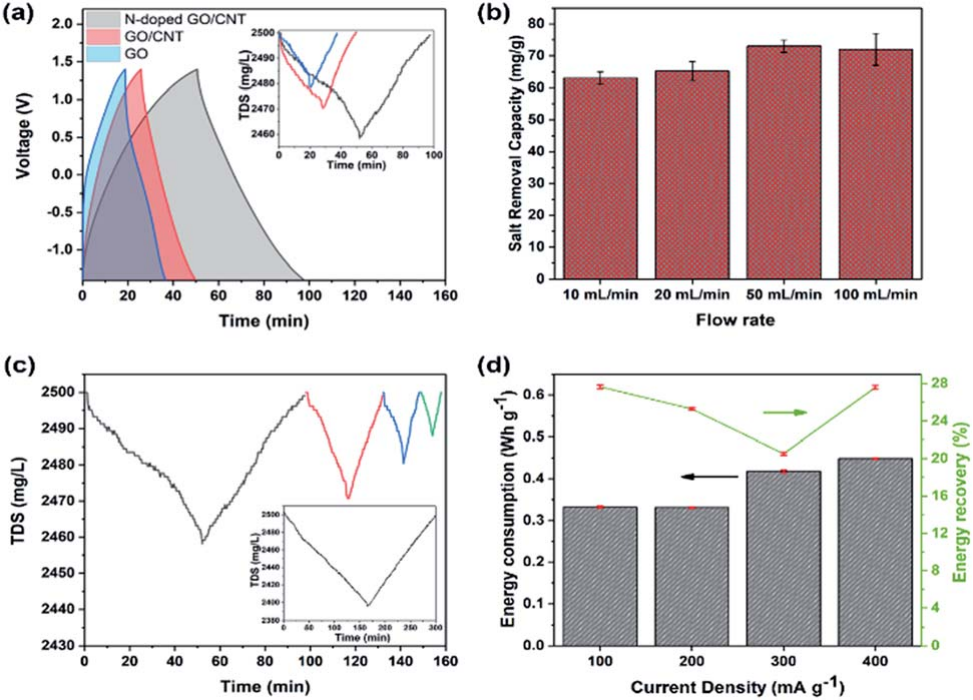
(a) The charge–discharge profile; the inset shows the corresponding total dissolved salt changes *vs.* time for three different 3D printed samples with the GO, GO/CNT, and NGO/CNT composition with the total electrode mass of around 20 mg, respectively. (b) Salt removal capacity for the different flow rates of 10 mL min^−1^, 20 mL min^−1^, 50 mL min^−1^, and 100 mL min^−1^ with the total electrode mass of around 20 mg. (c) Total dissolved salt changes *vs.* time for the different current densities of 100 mA g^−1^ (gray), 200 mA g^−1^ (red), 300 mA g^−1^ (blue), and 400 mA g^−1^ (green); the inset shows the TDS display for the higher total mass loading of 80 mg at 1 mA and the voltage range of −1.4–1.4 V. (d) The corresponding energy consumption and energy recovery for different current densities based on the 20 mg 3D printed NGO/CNT electrodes.

To demonstrate the importance of an optimized composition in a 3D printed structure for its application as an MCDI electrode, herein, three different samples were printed. The desalination experiments were performed in a NaCl solution with the fixed mass ratio of 1 : 1 for cathode : anode and the flow rate of 50 mL min^−1^. Herein, the loading mass for each side electrode was kept at around 10 mg, which could be achieved exactly based on the number of printed layers. The voltage window was kept as broad as −1.4–1.4 V to increase the removal capacity. As shown in Fig. 5(a), the experiments were repeated for 3D printed GO, GO/CNT, and N-doped GO/CNT. The higher capacity of the 3D printed GO/CNT electrodes as compared to that of the GO electrode is ascribed to the increase in the surface area and conductivity of the composites upon the introduction of the CNTs into the system. Importantly, the charge–discharge time of the GO/CNT is longer as compared to that of the GO electrode, indicating higher specific capacitance of the GO/CNT, which agrees with the CV results. It is noteworthy that there is a small deviation in the voltage profiles of all three types of electrodes, as observed in Fig. 5(a). This can be due to the low concentration of the electrolyte, which may lead to a small deviation from the ideal triangular shape due to insufficient ion concentration.^48^

It was found that the 3D printed N-doped GO/CNT showed largest total dissolved salt changes of 40 mg L^−1^. The performance enhancement could be due to different effects such as the effects of the doped nitrogen atoms and the corresponding developed defects and the higher specific capacitance.^44^ To further investigate the specific capacitance improvement, galvanostatic charge–discharge analysis was carried out for both the N-doped GO/CNT and GO/CNT electrodes at the four current densities of 0.1 A g^−1^, 0.2 A g^−1^, 0.5 A g^−1^, and 1 A g^−1^. As shown in Fig. S4(a) and (b),† the GCD curve obtained after nitrogen doping is significantly greater as compared to that of the primary ink. Moreover, the extended tail of the GCD curves after nitrogen doping is the result of the attachment of functional groups, originating from nitrogen sources, onto the surface of graphene; it should be noted that the capacitance obtained from the GCD analysis can be used as an estimation of the further desalination performance of the samples.^48^ Hence, based on the observed performances, the 3D printed N-doped GO/CNT was selected for the rest of the desalination experiments. The effects of flow rate on the salt removal capacity of the 3D printed N-doped GO/CNT were also studied. As shown in Fig. 5(b), a slight improvement in the desalination performance was observed upon increasing the flow rate, which was due to the facilitation of ion diffusion and convection effects in the structure of the electrodes.^8,49^ The rate capability of the MCDI system was examined at the different current densities of 100 mA g^−1^, 200 mA g^−1^, 300 mA g^−1^, and 400 mA g^−1^ with respect to the one side electrode mass, and the results are illustrated in Fig. 5(c). At higher current densities, the removal capacity of 75 mg g^−1^ decreased to 60 mg g^−1^, 40 mg g^−1^, and 20 mg g^−1^ at 100 mA g^−1^, 200 mA g^−1^, 300 mA g^−1^, and 400 mA g^−1^, respectively. This trend can be explained due to the incomplete absorption reaction as a result of fast charging/discharging processes at higher current densities.^8,13^ However, the removal capacity recovered to the initial value when the current density returned to the low value of 100 mA g^−1^. More desalination experiments were also performed with the higher total electrode mass loading of 80 mg to have a better comparison with other carbon-electrode MCDI results. Moreover, as shown in the inset of Fig. 5(c), the total dissolved salt changes of around 100 mg L^−1^ were achieved. In addition, a further comparative study on energy consumption and energy recovery as a function of current density was conducted, and the results are shown in Fig. 5(d). In brief, the energy consumption was obtained by integration of the charge–discharge voltage pattern with respect to the time multiplied by the applied current. The energy recovery in percentage was also measured using a previously discovered method.^8,14^ Based on the results, it was found that the energy consumption initially decreased, whereas it showed an obvious increase with a further increase in the current density. The energy recovery was lower (∼2%) at the higher current density of 200 mA g^−1^ as compared to that at 100 mA g^−1^. However, it should be noted that energy recovery is calculated exclusive of the salt removal capacity, and the values are calculated from the area under the charge–discharge voltage pattern. As a result, operating the system at the current density of 200 mA g^−1^ can be a comparable alternative to the case of 100 mA g^−1^, which leads to highest salt removal capacity.

To further gain an insight into the mechanism of salt ion removal, the concurrent changes in the voltage and total dissolved salt (TDS) patterns are illustrated in Fig. S4(c).† In the constant current operation mode, the electrodes were charged up to 1.4 V, and the sodium and chloride ions were simultaneously adsorbed on the anode and cathode, respectively; therefore, the concentration of brackish water would decrease accordingly. Once the maximum voltage of 1.4 V is reached, the current is reversed, and the ions are desorbed back into the feed solution according to the reverse polarization of electrodes. The experiments were repeated in the smaller voltage range of −1.2–1.2 V, and a decrease in the salt removal capacity was observed, as presented in Fig. S4(d);† the lower removal capacity as compared to that obtained in the wider voltage range was explained by an incomplete adsorption/desorption of the ions on the surface of the electrodes. Table 1 (ESI†) presents a comparative study with some of the previous carbon-based MCDI systems including their operational set-ups.

**Fig. 6:**
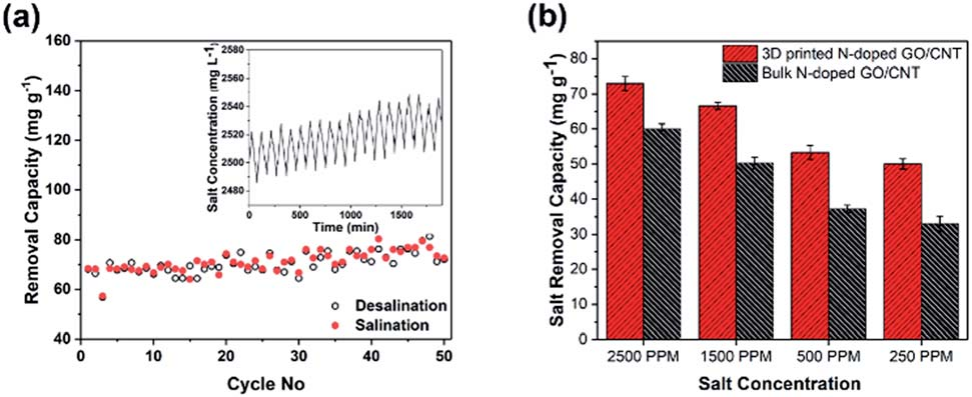
(a) The cycling performance of 3D printed N-doped GO/CNT electrodes during the cycling experiments with an initial solution concentration of ∼2500 ppm and the current density of 100 mA g^−1^. (b) The comparison of the salt removal capacity of 3D printed NGO/CNT and bulk NGO/CNT in solutions with different salt concentrations of 250 ppm, 500 ppm, 1500 ppm, and 2500 ppm.

In addition, the cycling stability of the electrodes as a crucial factor in the evaluation of the integrity of the electrode was studied by repeating the charging–discharging cycles. As observed in Fig. 6(a), the MCDI device consisting of the 3D printed N-doped GO/CNT electrodes showed excellent stability during 50 cycles, which could be attributed to the suitable mechanical stability along with the high conductivity of the final structure.^24^

To further shed light on the importance of the designed structure for the final desalination performance, a systematic study was performed to compare the bulk and 3D printed structures with the same composition. At first, real desalination experiments were conducted for both structures in solutions with different salt concentrations (Fig. 6(b)). As observed in Fig. 6(b), the different initial salt concentrations of 2500 ppm, 1500 ppm, 500 ppm, and 250 ppm were considered for the study. The average removal capacity decreased from 73 mg g^−1^ to 66 mg g^−1^, 53 mg g^−1^, and 50 mg g^−1^ and from 60 mg g^−1^ to 50.3 mg g^−1^, 37.3 mg g^−1^, and 33 mg g^−1^ for the 3D printed N-doped GO/CNT and bulk N-doped GO/CNT when the salt concentration was varied from 2500 ppm to 1500 ppm, 500 ppm, and 250 ppm, respectively. The higher removal capacity for the more concentrated salt solution was due to the improved mass transfer rate and the reduced electrical double layer thickness.^32^ However, for all the salt solution concentrations, the salt removal capacities are higher for the 3D printing structure as compared to that for the bulk sample. This can be attributed to the faster mass transfer and lower ion diffusion resistance due to the ordered macro channels in the designed 3D printed structure.^25^

**Fig. 7:**
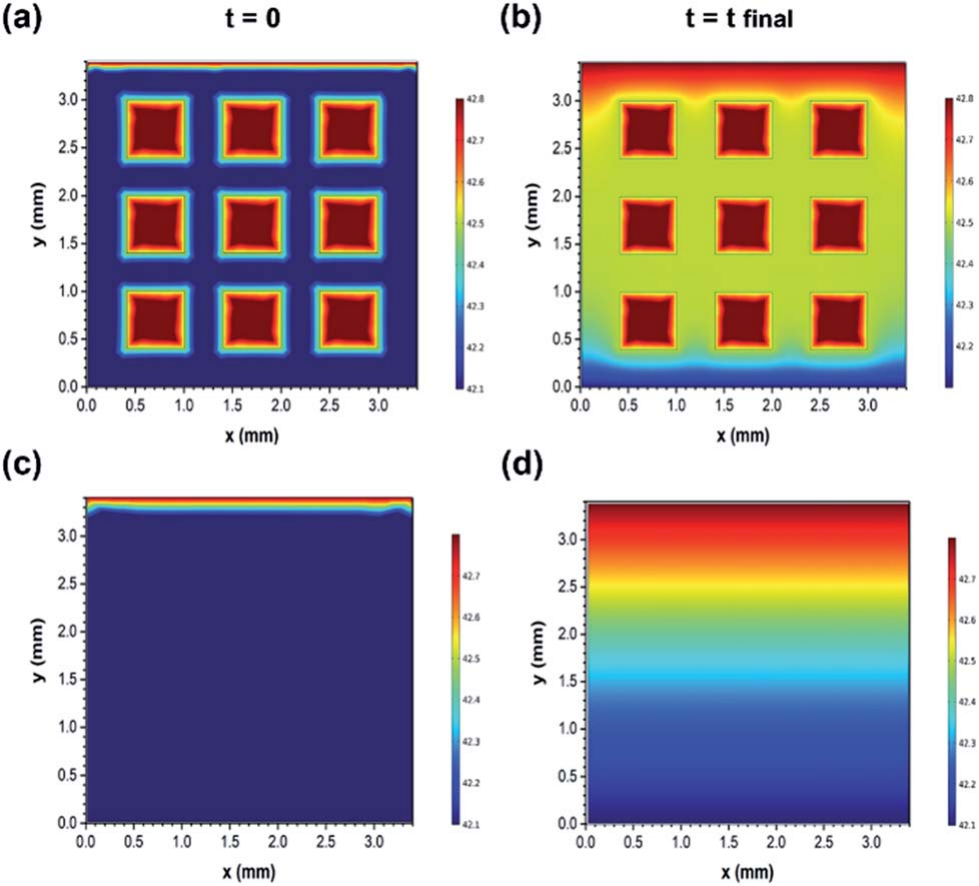
Simulation results for the constant concentration gradient between the inlet and outlet in two dimensions for time = 0 and time = *t*_final_. (a) and (b) 3D printed NGO/CNT; (c) and (d) bulk NGO/CNT. Color coding varies from red to blue for the highest (2500 ppm) to the lowest salt solution concentration (2460 ppm).

After this, the results were verified using the finite element simulations (Fig. 7(a)–(d)). Furthermore, the finite element simulation was conducted to evaluate the different ion transport behaviours of the two structures. The calculation was performed based on the constant gradient concentration between the inlet and outlet of the effective area of electrodes (3.4 mm × 3.4 mm). Both samples were speculated to have the same skeleton porosity consisting of micro/mesopores as they were prepared using the exact same methods. More simulation details are provided in the ESI.† As observed in Fig. 7(a)–(d), the variations in the ion concentration in the two dimensions of electrodes are illustrated for the initial (time = 0) and final (time = charging duration) time stages, and the faster ion diffusion in the 3D printed structure is clearly visible. In fact, macro holes can act as high concentration sources of ions and facilitate the distribution of sodium and chloride ions towards the micro/mesopores existing in the bulk of the electrodes. For instance, after the same charging time duration of 35 minutes, the average concentration (exclusive of macro hole concentration) of ions at the mid-height of the geometry (constant *y* value of 1.75 mm) reached up to 2485 ppm in the 3D printed structure, whereas it reached up to 2470 ppm for the bulk sample (the inlet concentration was fixed to 2500 ppm). Therefore, the simulation results are also in accordance with the experimental data and confirm the faster variation tendency of the ion concentration inside the 3D printed electrodes as compared to that in the bulk electrodes with the same composition.

## Conclusions

In summary, this study demonstrates a new and efficient approach for the fabrication of free-standing and scalable electrodes for membrane capacitive deionization devices. The final MCDI device consisted of two 3D printed nitrogen-doped GO/CNT electrodes, which improved the removal of the sodium and chloride ions from brackish salt solutions as compared to the bulk GO/CNT electrodes. The excellent mechanical stability and the optimized chemical composition of the electrodes facilitate the removal of salt ions and ensure the cycling stability of the assembled device. In addition, the influence of the 3D printed structure on the desalination performance of the electrodes was confirmed through experiments and finite element simulations. As a result, the proposed strategy can be extended to a variety of electrode compositions in different kinds of CDI devices (specifically HCDI by growing redox-active particles on 3D printed GO electrodes); moreover, it provides a promising means for increasing the likelihood of CDI commercialization as a cost-effective and energy-efficient technology for desalinating low to medium saltwater.

## Experimental

### Fabrication of 3D printing ink and 3D printed electrodes

The commercial GO solution with the concentration of 4 mg mL^−1^ was obtained from the Graphene Supermarket. Functional multi-walled CNT (with the length of 10–20 μm and the outer diameter of 30–50 nm) and ammonia hydroxide (NH_4_OH) were purchased from Time Nano, China, and Sigma Aldrich, respectively. The ink precursor was prepared through the dilution and mixing of a certain amount of GO : CNT (100 : 0, 95 : 5, 90 : 10, 85 : 15, and 70 : 30) followed by extended stirring for a few days to ensure the formation of a well-dispersed slurry. Subsequently, the mixture was fully dried using a freeze dryer (VirTis Benchtop Pro), and finally, viscous ink was obtained by redispersion in a small amount of water. The 3D electrodes were printed using a syringe dispenser (YS-D331-X Automatic Glue Dispenser). The syringe needles were fixed with a 30 GA gauge (the inner diameter of 0.006 inches), and the robotic arm was programmed to print simple cubic-like lattices with controlled layer numbers and the area of 16 × 16 mm on glass slides. The obtained product was freeze-dried, followed by subjection to a hydrothermal reaction in a Teflon-lined stainless-steel autoclave at 200 °C for 6 hours to reduce and dope the nitrogen atoms into the 3D printed GO/CNT electrodes.^43^ The hydrothermal set-up was composed of a 2 mL of NH_4_OH solution added to 20 mL deionized water, and the 3D printed sample was kept above the reaction solution using the Teflon column to prevent the direct contact with the solution. The well-maintained macrostructure was labeled as the 3D printed N-doped GO/CNT. The prepared electrodes were washed with deionized water to remove any impurity and kept for further characterization and desalination experiments after drying.

### Material characterization

The viscosities of the prepared inks were characterized using a stress-controlled rheometer (Discovery HR-2). A dynamic mechanic analyzer (TA Q800) was used to quantify the mechanical properties of the macrostructures *via* the compression stress (*σ*) responses of the samples to different strain (*ε*) percentages; a field-emission scanning electron microscope (FE-SEM, Jeol JSM-7600F) equipped with an energy dispersive X-ray spectrometer (EDS) was used to study the morphology of the final electrodes. The surface area of the electrodes was measured using the Brunauer–Emmett–Teller (BET) method (Autosorb-iQ-MP-XR), and the porosity distributions were derived from the density functional theory (DFT) method. The Raman spectra were obtained using the WITEC CRM200 Raman system (WITEC instruments Corp Germany, a 532 nm laser source). XPS was conducted using the Thermo Escalab 250Xi with an Al Kα source. The four-point probe station method was used to measure the conductivity of the electrodes (CMT-SR2000N). Cyclic voltammetry (CV) and galvanostatic charge–discharge (GCD) tests were carried out to evaluate the electrochemical performance of the samples using the three-compartment set-up composed of a working electrode (ink slurry coated on graphite sheets), reference electrode (Ag/AgCl), and counter electrode (platinum) in a 1 M NaCl solution (Bio-Logic VMP3, France electrochemical workstation). The specific capacitances of the prepared samples were calculated from their CV curves using the following equation:1
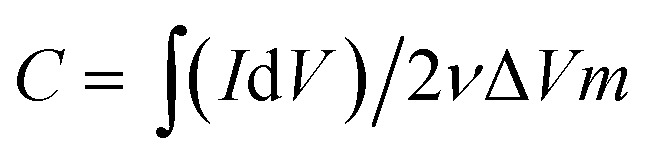
where *C* is the specific capacitance (F g^−1^), *I* is the responded current density (A), *V* is the applied voltage window (V), *ν* is the potential scan rate (V s^−1^), and *m* is the active material mass (g).

### Desalination experiments

The desalination system consisted of the novel 3D printed electrodes as the anode and cathode in a symmetric MCDI configuration. The experiments were carried out using an MCDI cell consisting of a cathode and an anode (3D printed N-doped GO/CNT) and an anion and a cation exchange membrane placed in front of the cathode and anode, respectively, and the spacer in the middle. The whole set up was tested in the constant current mode, and water was passed by the electrodes. The assembled MCDI device was connected to a battery analyzer (Neware, Shenzhen, China), a conductivity meter (DDSJ-308F, Leici), and a peristaltic pump. The conductivity of the outlet solution and the voltage variation were determined concurrently during the desalination experiments. The initial concentration of the 50 mL feed solution was varied from 250 ppm to 500 ppm, 1500 ppm, and 2500 ppm and the flow rate varied from 10 mL min^−1^ to 20 mL min^−1^, 50 mL min^−1^, and 100 mL min^−1^, respectively. The different current densities of 100 mA g^−1^, 200 mA g^−1^, 300 mA g^−1^, and 400 mA g^−1^ were examined for the rate capability study, and the cycling experiment was conducted at the current density of 100 mA g^−1^. The voltage range was chosen to be from −1.4 V to 1.4 V and from −1.2 V to 1.2 V, which were both within the water stability voltage window range. The salt removal capacity of the MCDI set-up was calculated according to eqn (2):

2 Salt removal capacity = (*C*_i_ − *C*_f_)*V*/*m*

where *C*_i_ and *C*_f_ are the initial and final concentrations of the NaCl solution for both the salination and desalination steps (mg L^−1^), *V* is the volume of the feed solution (L) and *M* is the total mass of the electrodes (g).

## Conflicts of interest

There are no conflicts to declare.

## Supplementary Material

NA-001-C9NA00507B-s001
