# Supplementary material for: 3D printed electrodes for efficient membrane capacitive deionization
Source: Nanoscale Adv. 2019 Oct 8;1(12):4804–11. doi: 10.1039/c9na00507b (PMC9418887; doi:10.1039/c9na00507b)
Supplement: NA-001-C9NA00507B-s001 [file NA-001-C9NA00507B-s001.pdf]

Electronic Supplementary Material (ESI) for Nanoscale Advances.
This journal is © The Royal Society of Chemistry 2019

Supplementary File

3D Printed Electrodes for Efficient Membrane Capacitive Deionization

Sareh Vafakhah,[a] Glenn Sim Joey,[a] Mohsen Saeedikhani,[b] Pablo-Valdivia y Alvarado,[a] Xiaoxia Li,[c] and Hui Ying Yang,*[a]

[a] Pillar of Engineering Product Development, Singapore University of Technology and Design, Singapore 487372

[b] Department of Materials Science and Engineering, National University of Singapore, 9 Engineering Drive 1, Singapore 117576

[c] Beijing Advanced Innovation Centre for Biomedical Engineering, School of Chemistry, Beihang University, Beijing 100191, P. R. China

*Corresponding Author

Email: yanghuiying@sutd.edu.sg

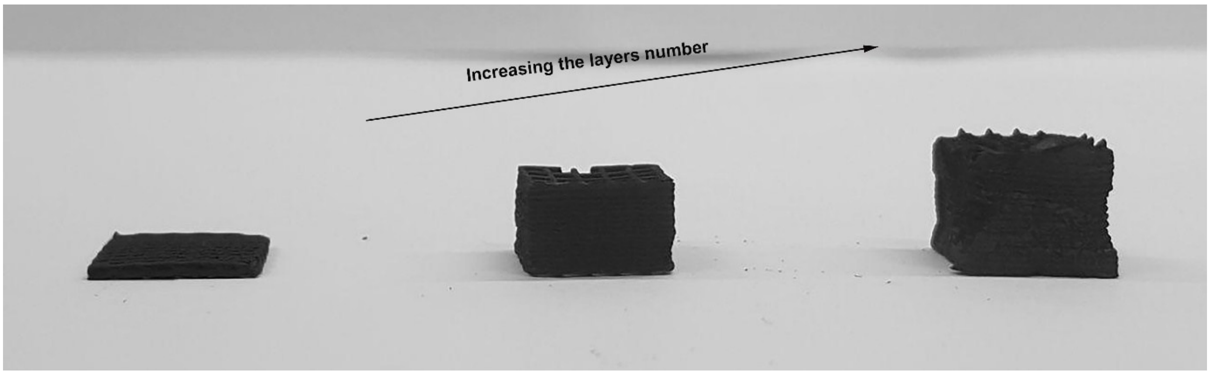

Figure S1, the photographs of the 3D printed GO/CNT with different number of layers.

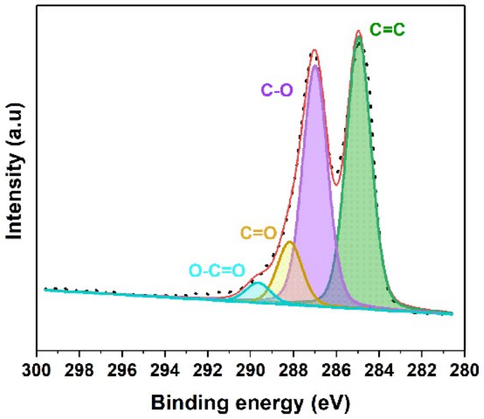

Figure S2, C1S spectra of 3D printed GO/CNT.

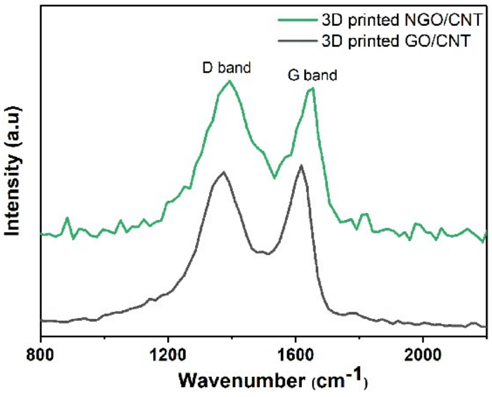

Figure S3, Raman Spectra of 3D printed GO/CNT before and after the nitrogen doping.

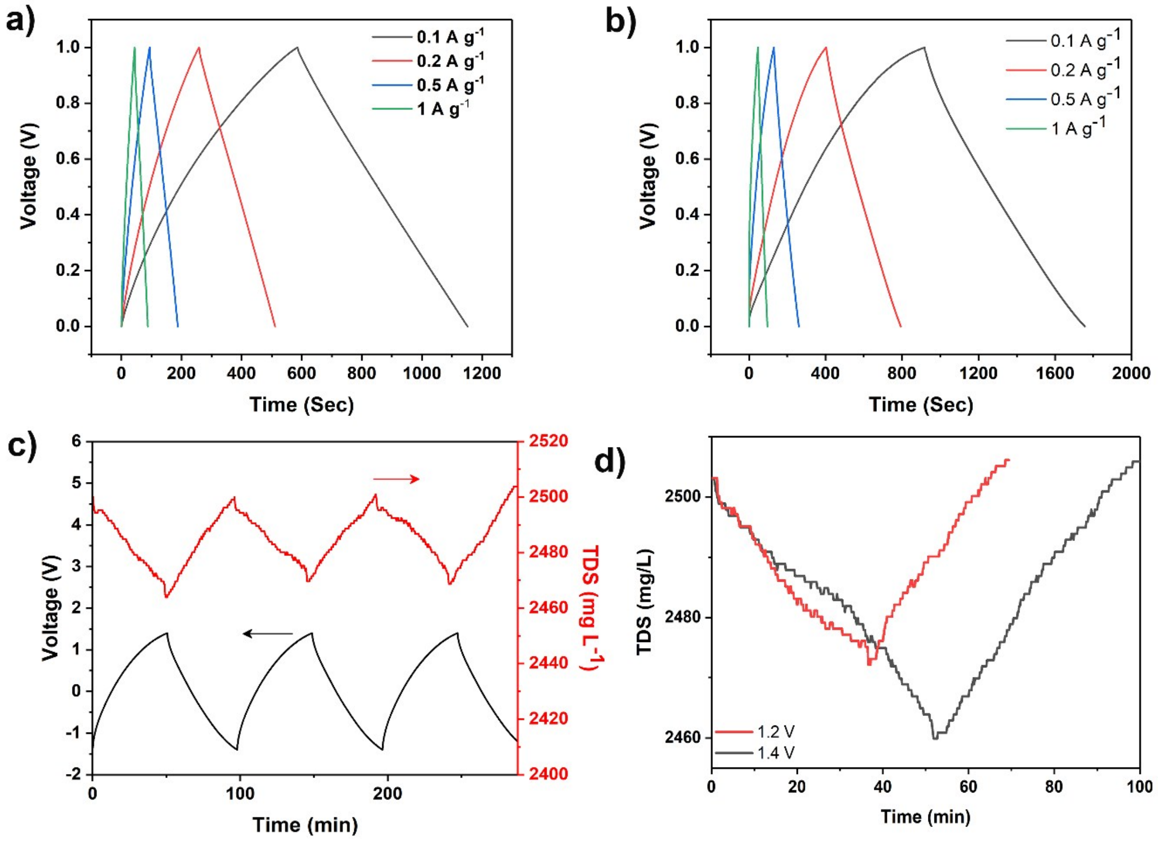

Figure S4, Galvanostatic charge-discharge curves for (a) GO/CNT 15% and (b) 3D printed Nitrogen doped GO/CNT 15% electrodes at different current densities. (c) Corresponding changes of voltage and total dissolved salt (TDS) of 20 mg 3D printed N-doped GO/CNT electrodes during the cycling experiments with an initial solution concentration of ~ 2500ppm and the current density of 100 mA g^{-1}. (d) Influence of voltage range on salt removal capacity in two different voltage ranges of -1.2V to 1.2V and -1.4 V to 1.4 V with total electrodes masses of 20 mg and current density of 100 mA g^{-1}.

Table 1. Comparison of Salt Removal Capacity of a few of current study for carbon-based materials in MCDI systems.

Electrode materials	Initial Salt solution concentration	Operational mode	Voltage/ Current density	Salt Removal Capacity (mg/g)	Electrodes mass	Reference
Activated Carbon	1000 mg/L	CC	1 V/ 1.43 mA/cm^2	15.7	650 mg	[1]
polyvinyl alcohol (PVA) on graphene oxide (GO)	750 mg/L	CV	1.2 V	30	120	[2]
porous carbon nanostructures (MOF derived)	750 mg/L	CV	1.4 V	45.62	90	[3]
Porous resorcinol-based mesoporous carbon	0.5 M	CV	2.4 V	64.7	-	[4]
Carbon aerogel	0.5 M	CV	2.4 V	43.4	-	[4]
Activated Luffa c arbon	2500 mg/L	CV	1.2 V	38	100	[5]
N-doped 3D printed GO/CNT	2500 mg/L	CC	1.4 V/ 25 mA/g	62	80	This work

Simulation:

The finite Element Modelling (FEM) of ion diffusion was performed using COMSOL Multiphysics. An arbitrary sample size of 3.4 mm × 3.4 mm was selected for the simulations and the geometry of the samples are depicted in Figures S4. For the both geometries the inlet's and outlet's concentrations (boundaries (2) and (4)) were set to 2500 ppm and 2460 ppm, respectively. Which are similar concentrations to the experiments. Moreover, boundaries (1) and (3) were considered insulators which means the flux of species are zero through these. In addition, for the 3D printed sample, boundaries (5) – (8) as well as the macro-holes (domain (β)) were assumed to have the concentration of 2500 ppm which means they are pre-filled with the same electrolyte as inlet. Furthermore, the structure for both samples was considered to have the same porosity as they have been prepared with the same method. The micro/meso-pores in both samples were assumed to be initially filled with 2460 ppm electrolyte.

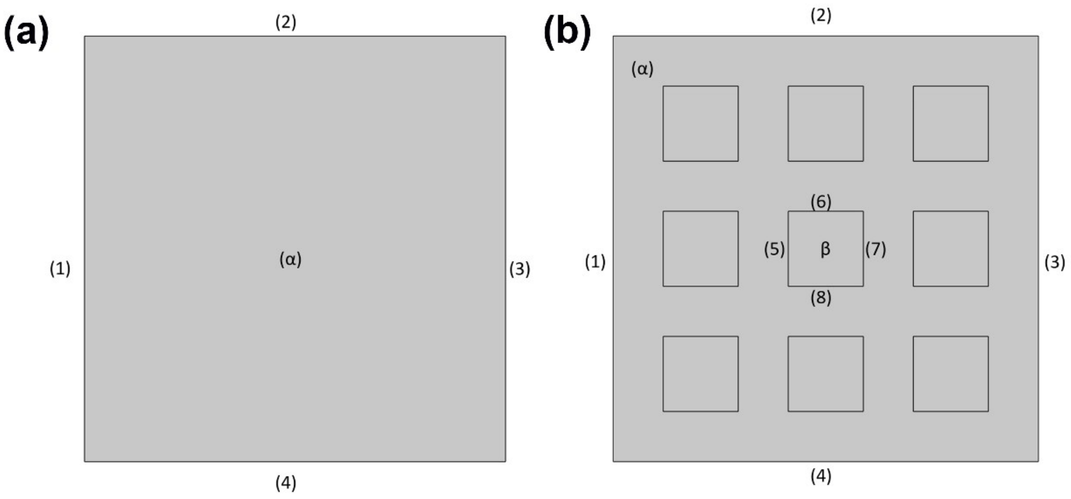

Figure S5, the geometries of the samples built in COMSOL Multiphysics software (a)Bulk N-doped GO/CNT, and (b) 3D printed N-doped GO/CNT.

Table 2 summarizes the boundary conditions:

Table 2, Boundary conditions of finite element simulation for both 3D printed and bulk N-doped GO/CNT electrodes.

Boundary	Description
(1) inlet	$c_i = 2500\ ppm$
(2) insulating	$-n.J_i = 0$
(3) insulating	$-n.J_i = 0$
(4) outlet	$c_i = 2460\ ppm$
(5)– (8) inlet concentration	$c_i = 2500\ ppm$

This simulation focuses on diffusion-controlled transport process. Therefore, Fick’s second law was applied on the computational domains (domain α in the 3D printed structure and the whole domain of the bulk sample):

$$\frac{\partial c_i}{\partial t} = D_0 \nabla^2 c_i$$

where c_i is the concentration of Na^+, $D_0 = 1.3 \times 10^{-9}\ [m\hat{}2/s]$ is the diffusion coefficient of Na^+ at 298 K, and $\nabla^2 c_i$ is the Laplace equation for the distribution of Na^+ in the electrolyte.

In addition, the Millington and Quirk model was used to correct the diffusivity in porous media:

$$\tau_{F,i} = \varepsilon_p^{-1/3}$$

where $\tau_{F,i}$ is tortuosity and ε_p is porosity.

1. J.-H. Choi and D.-J. Yoon, *Water Res.*, 2019, **157**, 167-174.
2. Z. Y. Leong, G. Lu and H. Y. Yang, *Desalination*, 2019, **451**, 172-181.
3. M. Ding, W. H. Shi, L. Guo, Z. Y. Leong, A. Baji and H. Y. Yang, *Journal of Materials Chemistry A*, 2017, **5**, 6113-6121.
4. K. Tang, Y.-h. Kim, J. Chang, R. T. Mayes, J. Gabitto, S. Yiacoumi and C. Tsouris, *Chem. Eng. J.*, 2019, **357**, 103-111.
5. D. Sriramulu, S. Vafakhah and H. Y. Yang, *RSC Adv.*, 2019, **9**, 14884-14892.
